# 1394. Antibiotics Overuse and Cost Analysis among Hospitalized Dengue Virus Infected-Thai Adults.

**DOI:** 10.1093/ofid/ofad500.1231

**Published:** 2023-11-27

**Authors:** Thundon Ngamprasertchai

**Affiliations:** Faculty of Tropical Medicine, Mahidol University, Bangkok, Krung Thep, Thailand

## Abstract

**Background:**

Dengue is a common cause of acute febrile illness, predominantly in Asia which required supportive treatment without antibiotics. The antibiotics overuse leads to antimicrobial resistance threats. This study aimed to determine the use of antibiotics and cost analysis of hospitalized dengue virus infected-Thai adults.

**Methods:**

This retrospective cohort study was conducted in Hospital for Tropical Diseases, Faculty of Tropical Medicine, Mahidol University, Thailand in 2022. All confirmed dengue adults’ cases during 2016-2021 were evaluated by two independent reviewers. Clinical, microbiological characteristics and medical costs per episode were interested outcome. The overuse was defined as inappropriate antibiotics prescription in terms of indications, choices, duration, dosage, or route administration. Factors associated with antibiotics overuse were evaluated using a Poisson regression. Direct medical and non-medical costs per episode also were analysed.

**Results:**

A total of 249 participants were included. More than half were classified as severe or dengue with warning signs upon admission. Nearly 20% were transferred to or admitted at intensive care unit. The cumulative incidence of antibiotics use was 9.3% (95% confidence interval (CI): 8.23–10.47), which majority were empirically given. Of 38.3% among empirical antibiotics given participants showed no definite concurrent bacterial infections. The cumulative incidence of antibiotics overuse was 49.8% (95% CI: 43.42–56.18) mainly thanks to no indications of antibiotics. There was association between disease severity upon admission and antibiotics overuse (*p*-value 0.014). Median total medical cost was 1,086.6 USD per episode; meanwhile, median cost of antibiotics use was 10.8 USD per episode. There was significant difference of antibiotics cost between overuse and appropriate use (*p*-value 0.005).

**Conclusion:**

The antibiotics overuse in hospitalized dengue virus infected-Thai adults was high. Doctors in charge should reconsider presumed concurrent bacterial infection before empirical antibiotics administration. Antimicrobial stewardship should be also endorsed in dengue cases or other tropical infectious diseases to control resistance threats and hospitalized medical costs.

**Disclosures:**

**All Authors**: No reported disclosures

